# Identification of a Novel Small Non-Coding RNA Modulating the Intracellular Survival of *Brucella melitensis*

**DOI:** 10.3389/fmicb.2015.00164

**Published:** 2015-03-19

**Authors:** Yufei Wang, Yuehua Ke, Jie Xu, Ligui Wang, Tongkun Wang, Hui Liang, Wei Zhang, Chunli Gong, Jiuyun Yuan, Yubin Zhuang, Chang An, Shuangshuang Lei, Xinying Du, Zhoujia Wang, Wenna Li, Xitong Yuan, Liuyu Huang, Xiaoli Yang, Zeliang Chen

**Affiliations:** ^1^Department of Laboratory Medicine, The General Hospital of Chinese People’s Armed Police Forces, Beijing, China; ^2^Department of Infectious Disease Control, Institute of Disease Control and Prevention, Academy of Military Medical Sciences, Beijing, China; ^3^Laboratory of Clinical Immunology in Jiangsu Province, Department of Clinical Laboratory, The First Affiliated Hospital of Soochow University, Suzhou, China; ^4^State Key Laboratory of Microbial Resources, Institute of Microbiology, Chinese Academy of Sciences, Beijing, China

**Keywords:** *Brucella*, small RNA, intracellular survival, post-transcriptional control, stress response, virulence

## Abstract

Bacterial small non-coding RNAs (sRNAs) are gene expression modulators respond to environmental changes, stressful conditions, and pathogenesis. In this study, by using a combined bioinformatic and experimental approach, eight novel sRNA genes were identified in intracellular pathogen *Brucella melitensis*. BSR0602, one sRNA that was highly induced in stationary phase, was further examined and found to modulate the intracellular survival of *B. melitensis*. BSR0602 was present at very high levels *in vitro* under stresses similar to those encountered during infection in host macrophages. Furthermore, BSR0602 was found to be highly expressed in the spleens of infected mice, suggesting its potential role in the control of pathogenesis. BSR0602 targets the mRNAs coding for *gntR*, a global transcriptional regulator, which is required for *B. melitensis* virulence. Overexpression of BSR0602 results in distinct reduction in the *gntR* mRNA level. *B. melitensis* with high level of BSR0602 is defective in bacteria intracellular survival in macrophages and defective in growth in the spleens of infected mice. Therefore, BSR0602 may directly inhibit the expression of *gntR*, which then impairs Brucellae intracellular survival and contributes to *Brucella* infection. Our findings suggest that BSR0602 is responsible for bacterial adaptation to stress conditions and thus modulate *B. melitensis* intracellular survival.

## Introduction

The survival of pathogens within a host is highly dependent upon their ability to sense and adapt to changes in the host environments. This entails a coordinated regulation of virulence genes in response to various environmental stresses. Recently, small non-coding RNAs (sRNAs) have attracted a great interest as important regulators in both eukaryotes and prokaryotes (Storz, 2002; Waters and Storz, 2009; Man et al., 2011). In different bacterial species, sRNAs may play crucial roles in the control of gene expression in regards to environmental changes, such as iron limitation, temperature shift, osmotic shock, envelope stress, nutrient stress, and metabolic imbalance (Toledo-Arana et al., 2007; Hoe et al., 2013). In addition, sRNAs have been postulated to mediate virulence gene expression in several pathogenic bacteria and their survival in hosts (Toledo-Arana et al., 2007; Papenfort and Vogel, 2010). The bacterial sRNAs are generally ranged from 50 to 300 nt in length. They are divided into cis-encoded sRNAs, trans-encoded sRNAs, protein binding sRNAs, and the recently discovered CRISPR sRNAs according to the mechanism used to control their targets (Waters and Storz, 2009). Of them, trans-encoded sRNAs is the best characterized and most extensively studied sRNAs, which could regulate gene expression by imperfect base-pairing with target mRNAs, thereby modulating mRNA translation and/or stability (Gottesman, 2004; Livny and Waldor, 2007; Papenfort and Vogel, 2010). This family of sRNAs is generally located in the “intergenic region” between protein-coding sequences. In Gram negative bacteria, the RNA binding protein Hfq is usually required to facilitate the interaction between trans-encoded sRNAs and their target mRNAs (Arnvig and Young, 2012).

*Brucella* spp. is facultative intracellular pathogenic α-proteobacteria that causes undulant fever, endocarditis, arthritis, and osteomyelitis in humans and abortion in domestic animals (Corbel, 1997; Godfroid et al., 2005). Unlike enteric pathogens that rely on the expression of specialized “virulence factors,” the virulence of Brucellae depends on their survival and replication abilities within host phagocytes (Kaufmann, 2011). Multiple genes associated with the intracellular trafficking and multiplication has been identified in *Brucella*. However, the complex post-transcriptional regulation and coordination of gene expression that enables *Brucella* to adapt to changes in their environment, evade host cell defenses, and survive in a hostile host environment, remains poorly understood. Taking into account, the obvious role of sRNAs as regulators associated with bacterial responses to stress, it is possible that sRNA play important roles in *Brucella* as well. *Brucella* strains deficient for Hfq, a protein usually required to facilitate sRNA–mRNA interactions, displayed extreme attenuation in mice and increased sensitivity to various environmental stress (Robertson and Roop, 1999), indicating sRNAs might have a regulatory function in the pathogen–host interactions during *Brucella* infection. Recently, Caswell et al. (2012) identified two sRNAs linked to virulence in *Brucella abortus*, suggesting the role of sRNA in *Brucella* pathogenicity. Identification of new sRNAs that regulate *Brucella* intracellular survival may provide insight into the pathogenesis and provide a new prospective in the fight against brucellosis.

In this text, we described the bioinformatics identification and experimental confirmation of novel identified sRNAs in *Brucella melitensis*. Furthermore, we systematically investigated the regulation mechanism of a novel sRNA BSR0602 modulating the intracellular survival of *B. melitensis*.

## Materials and Methods

### Bacteria, growth condition, and stress

*Brucella melitensis* strain 16M and its derivatives were routinely cultured in rich medium tryptic soy broth (TSB) at 37°C. *Escherichia coli* strain DH5α was grown on Luria–Bertani (LB) medium. Plasmid pBBR1MCS-4, a broad host range plasmid capable of replicating in *Brucella*, was kindly provided by Professor Kenneth M. Peterson (Kovach et al., 1995; Beckmann et al., 2010). Antibiotics were added to media when required at final concentrations of 50 μg/mL of kanamycin or 100 μg/mL of ampicillin. For stresses, *B. melitensis* 16M was grown in TSB to the middle exponential phase at 37°C, washed with PBS and then re-suspended as described below. To starve bacteria of nutrients, cells were re-suspended in GEM medium (MgSO4⋅7H_2_O 0.2 g/L, citric acid⋅H_2_O 2.0 g/L, K_2_HPO4 10.0 g/L, NaNH_4_HPO_4_⋅4H_2_O 3.5 g/L, glucose 20 g/L, pH 7.0) (Kulakov et al., 1997) at 37°C. To induce acid stress, cells were re-suspended in TSB broth (pH 4.0). To induce oxidative stress, H_2_O_2_ was added to the cultures at a final concentration of 1.5 mM at 37°C. To induce heat shock, cells were re-suspended in TSB broth at 42°C. As a control, a 50 mL culture was re-suspended in TSB broth at 37°C. Bacteria were incubated under various stress conditions for 30 min.

### RNA isolation

Total RNA was extracted from *B. melitensis* cultures using Trizol reagent (Invitrogen) as recommended by the manufacturer. Then, RNA samples were treated with DNAse I (Promega) to eliminate contaminating genomic DNA. RNA quantity and quality were assessed using ND-1000 Spectrophotometer Nanodrop (Technologies) and agarose gel electrophoresis.

### Northern blot

Northern blot analyses were carried out using a DIG northern starter kit (Roche) according to the manufacturer’s protocol as described previously (Beckmann et al., 2010; Deng et al., 2012). Briefly, total RNA (20 μg/sample) was denatured at 70°C for 5 min, separated on 10% polyacrylamide-7 M urea gel and then transferred to Hybond N^+^ membranes (GE) via electroblotting. The membranes were UV-cross-linked and prehybridized for 45 min, and 3′-end DIG-labeled RNA probes were added. The membranes were then hybridized overnight at 68°C in a DIG Easy Hyb according to the manufacturer’s protocols.

### 5′ and 3′ RACE

5′ and 3′ RACE was carried out using a Full RACE Core set (Takara Biochemicals) as recommended by manufacturer’s instructions. Prior to initiating the 3′ rapid amplification of cDNA ends (RACE) protocol, total RNA was polyadenylated by treatment with poly(A) polymerase (Ambion) at 37°C for 1 h. The PCR products were cloned into pMD19-T Vector (Takara Biochemicals), and then the clones were sequenced and analyzed. For each RACE analysis, 6–10 clones were sequenced, and the farthest 5′ (3′) end was considered as the 5′ (3′) end of the sRNA.

### Quantitative RT-PCR

The expression profiles of BSR0602 under *in vitro* environmental stress were compared by quantitative RT-PCR (qRT-PCR). Samples were amplified in a 25 μL volumes containing 12.5 μL of 2× SYBR Green I Master Mix (Takara Biochemicals), 100 nM each primer, and 1 μL of cDNA sample. Thermocycling conditions were as follows: 10 min at 95°C for pre-incubation, and then 45 cycles of amplification (95°C for 30 s, 60°C for 30 s, and 72°C for 30 s). The primers used for qRT-PCR are listed in Table S1 in Supplementary Material. All primer sets showed standard curves with *R*^2^ values of >0.980, 90–110% reaction efficiencies, and only one peak in dissociation curves. Relative transcriptional level was determined by the methods of 2^−ΔΔCt^ as described previously (Wang et al., 2009; Cui et al., 2013). The level of 16S rRNA was used as a reference gene to normalize the expression data for target gene. The average expression levels and SD were calculated using data from three technical replicates of three independent experiments.

For transcription analysis during mouse infection, 6- to 8-week-old female BALB/c mice (five per time point) were infected intraperitoneally with 2 × 10^6^ CFU of *B. melitensis* 16M. At 3, 7, 14, 28, and 42 days following infection, mice were sacrificed by cervical dislocation, spleens were removed aseptically and total RNA was isolated using the Trizol (Invitrogen) extraction method. Further qRT-PCR analysis was carried out as described above.

### Semi-quantitative RT-PCR

The TargetRNA program was used to predict the target mRNAs of BSR0602 against the entire genome of *B. melitensis* 16M (Tjaden et al., 2006) and the predicted mRNA targets of BSR0602 were validated by simi-quantitative RT-PCR as described previously (Wang et al., 2009). 16S rRNA was used as internal control. Different cDNA samples were amplified with primers for 16S rRNA, and the cDNA samples were normalized by differential dilutions according to quantity of 16S rRNA products. Then, selected genes were amplified from normalized cDNA samples with specific primers (Table S1 in Supplementary Material). The PCR products were analyzed on 1.2% agarose gel and visualized by ethidium bromide staining.

### Two-plasmid system for assessing target regulation by BSR0602

The *E. coli*-based system for studying sRNAs gene regulation developed by Urban and Vogel (2007) was used to assess the regulation of target mRNAs by *Brucella* BSR0602 according to a previously published protocol (Caswell et al., 2012). The genes encoding BSR0602 or a nonsense sRNA were amplified by PCR using genomic DNA from *B. melitensis* 16M as a template, Platinum^®^ Pfx DNA Polymerase (Invitrogen), and the primer sets BSR0602-express-For/Rev or nonsense sRNA-express-For/Rev, respectively. The amplified DNA fragments were digested with *Xba*I and treated with polynucleotide kinase, and the digested/treated fragments were then cloned into a derivative of pZE12 as described previously (Urban and Vogel, 2007). To construct the *gfp* fusion constructs, the regions from the 5′-UTR to the first 15 codons of a target *Brucella* gene was amplified by PCR using genomic DNA from *B. melitensis* 16M as a template, Platinum^®^ Pfx DNA Polymerase (Invitrogen), and specific primers (Table S1 in Supplementary Material). The PCR-amplified DNA fragments were digested with *Nsi*I and *Nhe*I, and ligated into pXG10-SF (Urban and Vogel, 2007). The authenticity of all constructs was confirmed by DNA sequence analysis. *E. coli* TOP10 cells (Invitrogen) were transformed either with a target *gfp* fusion plasmid alone or with a combination of a target *gfp* fusion plasmid and a sRNA expression plasmid. The levels of GFP in the *E. coli* strains were assessed by immunoblot analysis using the methods described previously (Corcoran et al., 2012). Here, GFP was detected using anti-GFP antibodies (Sant Cruze), and detection of GroEL as a loading control was performed with anti-GroEL antibodies (Enzo life science). Constructs expressing mutated BSR0602 and its target gene carrying point mutation were generated by a PCR-based mutagenesis approach.

### Generation of mutant and overexpression strains

The BSR0602 and *gntR* deletion strains were generated by resistance gene replacement as described previously (Cui et al., 2013). Approximately 500 bp sequences of each of the upstream and downstream regions of BSR0602 coding region were assembled in pUC19K (Wang et al., 2011) to generate suicide plasmid pUC19K-BSR0602. Similarly, the suicide plasmid pUC19K-gntR was constructed. The suicide plasmids were introduced individually into *B. melitensis* 16M and potential deletion mutants were isolated by their amp^S^ kan^R^ phenotype. The deletion mutant strains were confirmed by PCR amplification with primer pUC19K-F and BSR0602-I-R or pUC19K-F and BMEI0106-I-R, which located in kanamycin gene and downstream of homologous arm of BSR0602 or BMEI0106, respectively. The deletion mutants were further confirmed by RT-PCR.

The overexpression strain of BSR0602 was constructed by amplifying the wild-type BSR0602 locus using primers BSR0602-N-F and BSR0602-C-R from *B. melitensis* 16M and cloning it into the *Kpn*I-*Pst*I sites of pBBR1MCS4, a plasmid that could replicate in *Brucella*. The resulting plasmid pBBR-BSR0602 was electroporated into 16M, resulting in the overexpression strains 16M-BSR0602. The overexpression of BSR0602 was further confirmed by RT-PCR. Meanwhile, 16M-MCS, a derivative strain of 16M with the empty vector pBBR1MCS4, was used as the negative control strains.

The complementary strain of *gntR* was constructed by amplifying the wild-type BMEI0106 locus using primers BMEI0106-N-F and BMEI0106-C-R from *B. melitensis* 16M and cloning it into the *Kpn*I-*Pst*I sites of pBBR1MCS4 as above. Then, the resulting plasmid pBBR1-gntR was electroporated into 16MΔgntR, resulting in the complementary strain 16MΔgntR-C. The transcription restoration of *gntR* in the complementary strain was further confirmed by RT-PCR.

### Stress resistance assays

The susceptibility of *B. melitensi*s 16M, 16MΔBSR0602, and 16M-BSR0602 to various *in vitro* environmental stress conditions were determined as described previously (Cui et al., 2013). *B. melitensis* strains were first grown to stationary phase (OD_600_ = 2.5) at 37°C in TSB medium. The effects of various stresses were tested as follows: to determine the effect of high-salinity or high-osmolarity stress, the cells were incubated at 37°C for 20 min in the presence of NaCl (1.5 M); for acidification stress, the cells were incubated at 37°C for 15 min in TSB medium at pH 3.0; for oxidative stress, the cells were incubated at 37°C for 40 min in the presence of 440 mM H_2_O_2_; for heat shock, the cells were transferred to pre-warmed 50°C tubes and incubated at 50°C for 60 min. After treatment, cells were diluted and plated on TSA plates to determine viability. Results are expressed as a mean percentage ±SD from three independent experiments.

### Macrophage survival assay

Murine macrophage-like RAW264.7 were used to assess survival capability of 16MΔBSR0602 mutant, 16M-BSR0602 and their wild type strain 16M. In brief, monolayers of macrophages were seeded in 24 well plates 1 day prior to infection at 5 × 10^5^ cells per well. Wild type and mutant strains or wild type and overexpression cells obtained at mid-log phase were mixed in a 1:1 ratio to generate the inoculum (2.5 × 10^7^ CFU) for competition assays. Macrophages were infected with bacterial suspension at a MOI of 50. At 45 min post-infection, the cells were washed three times with PBS and then incubated with 50 μg/mL of gentamycin for 60 min to kill extra-cellular bacteria. The cultures were then replaced with DMEM with 20 μg/mL of gentamycin. At 4 and 24 h post the infection, the supernatant was discarded and cells were lysed, and the live bacteria were enumerated by plating in duplicate on TSA plates with or without kanamycin or ampicillin. Colony counts on plates containing antibiotics represent kanamycin-resistant ΔBSR0602 mutant or ampicillin-resistant BSR0602 overexpression strains, and these values were subtracted from the colony counts determined on plates representing both the mutant and the wild-type strains. Data are presented as a log_10_ value of CFU averaged over five wells.

### Competitive infections in mice

Groups of ten 6- to 8-week-old female BALB/c mice were infected intraperitoneally with an inoculum (2 × 10^6^ CFU/mL) representing a 1:1 ratio of wild-type *B. melitensis* 16M to mutant or overexpression strains. At 24 h post the inoculation, the infected mice were sacrificed by cervical dislocation and spleens were removed aseptically and homogenized with PBS containing 0.1% Triton X-100. Serial dilutions of spleen homogenates were prepared and plated in duplicate on TSA plates with or without kanamycin or ampicillin, and the CFU were counted after 4 days of infection at 37°C. The competitive index (CI) values were calculated from the ratios of total input and recovered wild-type and kanamycin-resistant ΔBSR0602 mutant (or ampicillin-resistant BSR0602 overexpression strains) CFU as previously described (Shea et al., 1996).

### Statistical analysis

Bacterial survivals under *in vitro* stresses and during *in vivo* infections were expressed as the mean percent of survival compared to untreated controls ±SD. Statistical analysis was performed using Student’s unpaired *t* test. For the CI assays, the data was analyzed by Student’s *t* test. For qRT-PCR experiments, significance was calculated by the Wilcoxon signed-rank test. In all cases, a *P* value of >0.05 was considered significant.

### Ethics statement

All animal experiments were performed in strict accordance with experimental animal regulation ordinances defined by China National Science and Technology Commission. The protocol was approved by Animal Ethics Committee of Beijing Institute of Disease Control and Prevention. Animals are provided with humane care and health conditions during their stay in the facility. All individuals who use animals receive instruction in experimental methods and in the care, maintenance and handling of mice, and are under the committee’s supervision.

## Results

### Computational predictions of candidate sRNAS in *B. melitensis*

Intergenic sequences were extracted from the *B. melitensis* 16M chromosome (NC_003317.1 and NC_003318.1) based on genome annotation. The intergenic regions (IGRs) with a minimal size of 80 bp were then subjected to BLAST against all *Brucella* spp. genomic sequences available at the NCBI web site. Hits with an *E* value <0.001 were thought to be *B. melitensis* specific and thus exclude from further analyses. The replication origin, putative pseudogenes, and intergenic sequences encoding tRNAs or rRNAs were also excluded. Promoters were identified in IGRs with pftools 2.3^1^ with a cut-off value of 255, while terminators were identified using RNAMotif (Lesnik et al., 2001). Motif descriptor came from sRNAPredict (Waldor Lab, Tufts University). IGRs with both promoters and terminators were defined as containing a possible sRNA gene.

Based upon these analysis criteria, a total of 21 candidate sRNA genes were predicted. These sRNA candidates were named BSR for “*Brucella* small non-coding RNA,” followed by the gene number of the adjacent downstream protein-coding gene. The sequences of these sRNA candidates were conserved in all *Brucella* species. Furthermore, it seems that these sequences could not encode small peptides as they lacked appropriately positioned start and stop codons. The genomic coordinates in *B. melitensis* 16M, the transcription orientation, flanking genes, and predicted lengths of these sRNAs candidates are shown in Table 1.

**Table 1:** **Putative sRNA-containing intergenic regions examined by northern blot analysis**.

No.	Predicted sRNA	Chromosome	Gene orientation	Coordinates in 16M genome (5′–3′)	Flanking gene		Predicted length (nt)	Probe location^a^
					5′	3′	
1	BSR1944^b^	AE008917	← ← ←	2004088-2003970	BMEI1945	BMEI1944	119	2–100
2	BSR0742	AE008918	→ ← →	781743-781583	BMEII0743	BMEII0742	161	26–133
3	BSR0709^b^	AE008918	← ← →	750924-750608	BMEII0710	BMEII0709	317	159–289
4	BSR0653^b^	AE008918	→ ← →	684479-683838	BMEII0654	BMEII0653	642	127–307
5	BSR1350^b^	AE008917	← ← →	1405266-1405091	BMEI1351	BMEI1350	176	33–138
6	BSR1007^b^	AE008917	← ← ←	1047074-1046906	BMEI1008	BMEI1007	169	5–144
7	BSR0743^b^	AE008917	→ → →	769464-769559	BMEI0742	BMEI0743	96	3–74
8	BSR0739^b^	AE008917	← → →	764954-765113	BMEI0738	BMEI0739	160	20–121
9	BSR0617^b^	AE008917	→ → →	643141-643300	BMEI0616	BMEI0617	160	31–143
10	BSR1073^b^	AE008918	← → ←	1114550-1114745	BMEII1072	BMEII1073	196	33–134
11	BSR0322^b^	AE008917	→ → ←	330458-330586	BMEI0321	BMEI0322	129	2–119
12	BSR0201^b^	AE008917	← → →	208543-208625	BMEI0200	BMEI0201	83	15–77
13	BSR1915	AE008917	→ → ←	1969915-1970035	BMEI1914	BMEI1915	121	5–112
14	BSR0742	AE008918	→ → →	778863-779065	BMEII0741	BMEII0742	203	4–142
15	BSR0626^b^	AE008918	← → ←	661282-661409	BMEII0625	BMEII0626	128	19–118
16	BSR0602^b^	AE008918	← → ←	635956-636124	BMEII0601	BMEII0602	169	33–142
17	BSR1141^b^	AE008917	← → ←	1187552-1187749	BMEI1140	BMEI1141	198	41–176
18	BSR0437^b^	AE008918	→ → →	456155-456421	BMEII0436	BMEII0437	267	12–227
19	BSR1133	AE008917	← → ←	1176626-1176882	BMEI1132	BMEI1133	257	47–155
20	BSR0377	AE008918	← → ←	392479-392638	BMEII0376	BMEII0377	160	8–139
21	BSR0992	AE008917	← → ←	1034662-1034877	BMEI0991	BMEI0992	216	60–159

*^a^The probe location is given relative to the start nucleotide of predicted sRNA, taken as +1*.

*^b^sRNA candidates experimentally confirmed by northern blot*.

### Experimental verification and expression profiles of sRNAS in *B. melitensis*

**Figure 1:**
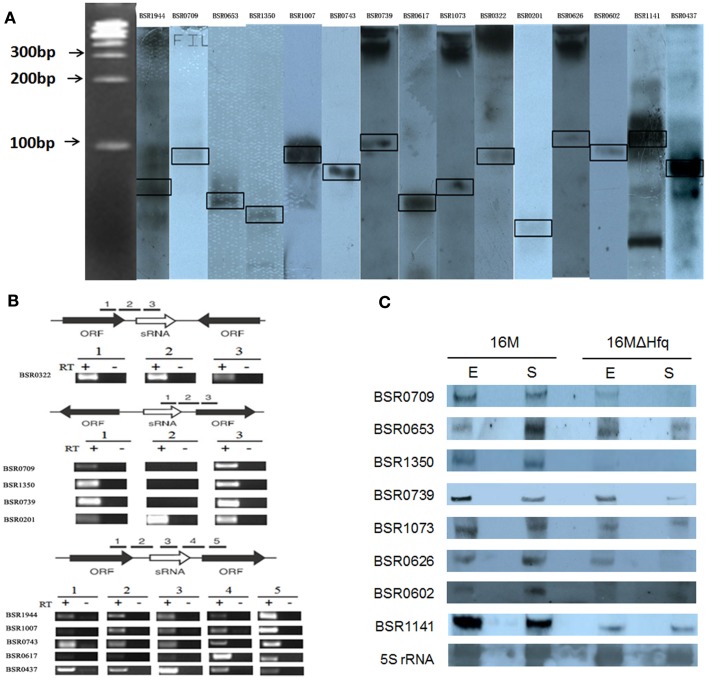
**Experimental verification and expression of the sRNAs in *B. melitensis***. **(A)** Northern blots verification for the presence of *B. melitensis* sRNAs. RNA from stationary phase cultures was analyzed by northern blotting using 3′-end DIG-labeled RNA probe complementary to sRNA candidates. **(B)** RT-PCR verification of the transcriptional unit of sRNA candidates. RNA prepared from wild-type cells grown to stationary phase at 37°C was used for the RT-PCR reaction. The gene organizations around sRNA genes were classified into three groups as schematically represented; the positions and directions of sRNAs and ORFs were represented by white and black arrows, respectively. The regions to be amplified were shown by bars with numbers. +, with reverse transcriptase; −, without reverse transcriptase. **(C)** Examination of sRNA expression in *B. melitensis* 16M and 16MΔhfq with northern blot. Total RNA was extracted from the 16M and 16MΔhfq grown in TSB to exponential phase (E) and early stationary phase (S). Northern blot was performed as described in Section “Materials and Methods.” 5S rRNA was used as a positive control. For each sRNA, northern blot analysis was carried out using at least three different RNA samples to ensure the reproducibility of expression profiles.

Northern blot hybridization was employed to verify the presence of these 21 putative sRNA candidates. Total RNAs were isolated from *B. melitensis* 16M grown in TSB7.0 at 37°C in exponential and stationary phases. For each putative sRNA region, a 3′-end DIG-labeled RNA probe was prepared for the most highly conserved portion of the sequence. Among 21 IGRs thus analyzed, 15 were reproducibly found to express transcripts (Figure 1A). RT-PCR results showed that all these 15 sRNAs were also present in other *Brucella* strains (Data not shown). The remaining six sRNAs candidates could not be detected by northern blot analysis, possibly resulted from that these candidate sRNAs were probably expressed at very low levels or not expressed under the present conditions.

Of the 15 sRNA candidate genes, the direction of BSR0653, BSR1073, BSR0626, BSR0602, and BSR1141 were opposite to the 2 flanking ORFs, suggesting that these 5 sRNA candidates could not be co-transcribed with the flanking ORFs. As expected, RT-PCR showed that the five sRNA candidates were transcribed independently. Then, the remaining 10 sRNA candidates were examined whether to be co-transcribed with their upstream and/or downstream genes. Using primer combinations that located in the sRNA and the adjacent gene, no products were observed for BSR0709, BSR1350, and BSR0739, indicating that they were transcribed independent of flanking genes (Figure 1B). The other seven sRNA genes were co-transcribed with the upstream and/or downstream genes, suggesting the possibility that these sRNAs may be part of or overlap with the 5′/3′ UTR of the adjacent ORF. Although we did not rule out the possibilities that these seven sRNA genes had their own promoters and they encoded sRNAs that overlap with the adjacent mRNAs, we excluded these seven sRNAs from the present sRNA list. Hence, we experimentally confirmed eight sRNAs in *B. melitensis*.

Similarity analysis performed with BlastN showed that the sequences of BSR1350, BSR0602, and BSR1141 were conserved in at least one related α-proteobacterium. To determine whether these eight sRNAs are newly identified, the sequences were blasted to the Rfam database^2^ and small RNA database^3^. Results demonstrated that none of these *B. melitensis* sRNA candidates had similarities with already identified sRNAs, indicating they are potentially novel identified sRNAs.

All the eight identified sRNAs genes were detected throughout the growth phase and five sRNAs displayed a growth-phase dependent expression profile (Figure 1C). BSR0739 and BSR0709 were abundant in the exponential growth phase, and decreased in stationary-phase cells. While the expression level BSR0653, BSR0626, and BSR0602 were higher in stationary phase than that in the exponential phase. The other three sRNAs are likely to be growth-phase independent for their expression levels appeared to be unchanged.

In many cases, trans-encoded sRNAs usually requires the RNA chaperone protein Hfq, which facilitates the interaction between sRNAs and target mRNA (Storz et al., 2004; Valentin-Hansen et al., 2004). To determine whether these eight verified sRNAs is associated with Hfq, we compared the expression level of these sRNAs in an *hfq* mutant and the isogenic wild-type strain by northern blot hybridization. The expressions level of six sRNAs (BSR0709, BSR1350, BSR0739, BSR0626, BSR0602, and BSR1141) were affected in the Δhfq mutant, particularly, transcriptional levels of BSR1350, BSR0602, and BSR1141 was significantly decreased when Hfq was inactivated, indicating that the expression or stability of these sRNAs was dependent on Hfq (Figure 1C).

### BSR0602 is induced *in vitro* stress conditions and during *in vivo* infection

**Figure 2:**
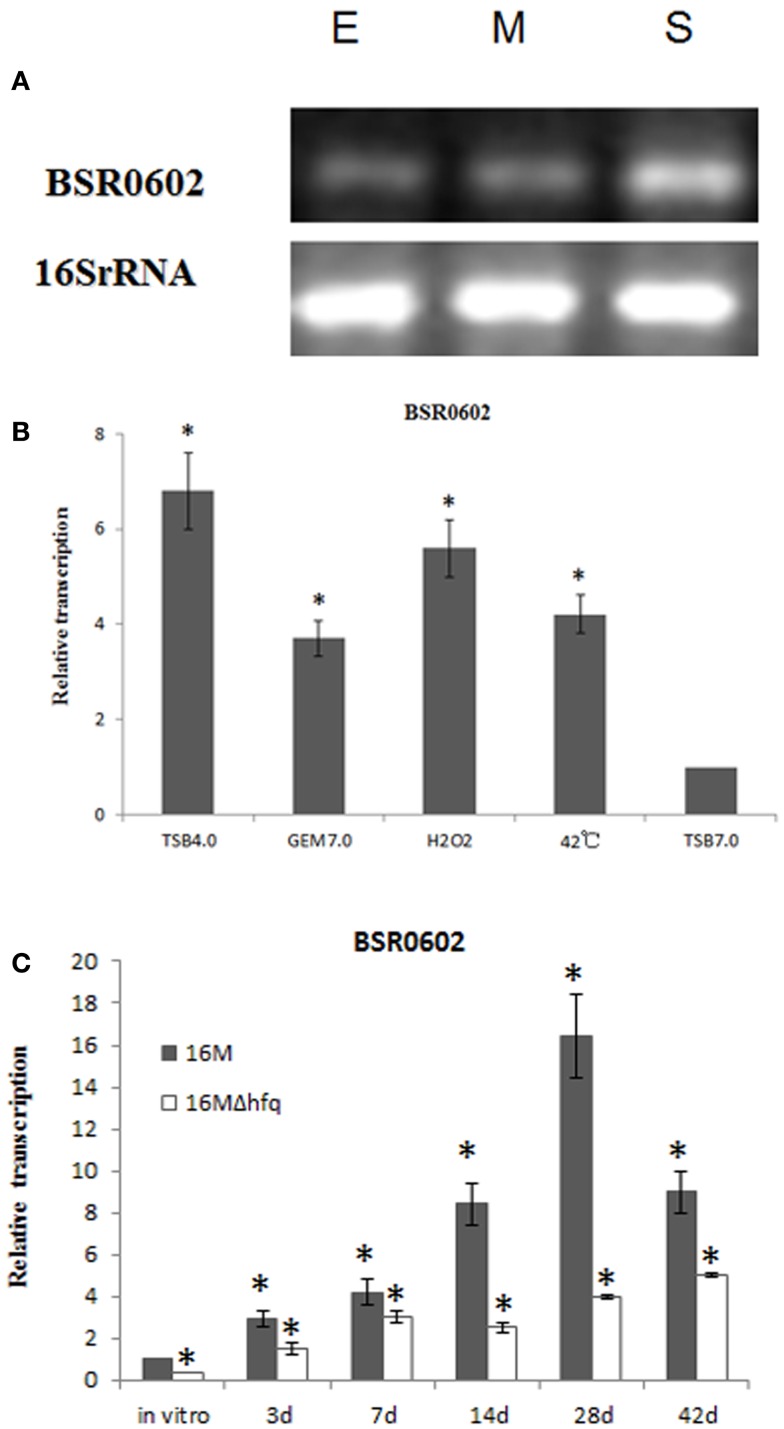
**BSR0602 is expressed under infection related conditions**. **(A)** Growth dependent expression of BSR0602. Total RNA was extracted from *B. melitensis* 16M grown in TSB to early-exponential stage (E), mid-exponential stage (M), and early stationary phase (S). RNA was isolated and transcription of BSR0602 was quantified by semi-quantitative RT-PCR. The 16S rRNA was used as an internal control. **(B)** Expression of BSR0602 *in vitro* environmental stresses. 16M was firstly cultured in TSB (pH7.0) and then subjected to different *in vitro* environment stresses. RNA was isolated and transcription of BSR0602 was quantified by qRT-PCR. The values of the relative expression, which are the means from triplicate experiments, represent the ratios of the levels of BSR0602 under different *in vitro* stresses to that under the regular TSB7.0 condition. Asterisks (*) represent significant differences compared with that under TSB7.0. **(C)** Expression of BSR0602 during mouse infection. BALB/c mice (five per time point) were intraperitoneally infected with *B. melitensis* 16M and 16MΔhfq, and then the intracellular bacteria were recovered from the spleens at 3, 7, 14, 28, and 42 days post infection, respectively. Total RNA was isolated and subjected to qRT-PCR as described. The values represent the relative level of BSR0602 in 16M or 16MΔhfq recovered from the spleen as compared to the level of BSR0602 in *B. melitensis* grown to exponential phase *in vitro*. The asterisks above gray box denote values significantly different from those of *in vitro* condition, and asterisks above white box represent significant differences between 16M and 16MΔhfq.

BSR0602 transcript was produced abundantly in the stationary phase (Figures 1C and 2A), implying that it might play an essential role in the capacity of the *Brucellae* to establish and maintain long-term intracellular residence in host macrophages (Roop et al., 2003). Thus, BSR0602 was chosen for further function analysis. Firstly, the precise start and endpoints of BSR0602 was determined by using 5′ and 3′ RACE. Results showed that BSR0602 was 169 nt in length, and located in clockwise orientation at bps 635956-636124 of chromosome II of *B. melitensis*. The secondary structure of BSR0602 was predicted by using the MFOLD program (Zuker, 2003).

As intracellular bacterial pathogens, *Brucella* species can survive and replicate in host phagocytes, where they likely encounter different stresses such as oxidative stress, low pH, and limited nutrition (Teixeira-Gomes et al., 2000). To study the possible role of BSR0602 in *B. melitensis* intracellular survival, we investigate the expression profile of BSR0602 under stresses resembling those encountered during infection. The cultures of *B. melitensis* was subjected to nutrition limitation (GEM 7.0), acid stress (TSB 4.0), heat shock (induced by high temperature), oxidative stress (induced by H_2_O_2_), and standard *in vitro* growth condition (TSB7.0). Total RNA was isolated, and then the expression of BSR0602 under environmental stress was examined using qRT-PCR. Acidic pH condition is an environmental stress that *Brucella* encounters in host macrophages, and it could trigger an expression response required for successful adaptation to the intracellular environment. Exposure of *B. melitensis* to pH 4.0 (TSB4.0) for 30 min resulted in increased transcription of BSR0602 (Figure 2B). Oxidative stress, another important characteristic of the environment in the host phagocytes, induced accumulation of BSR0602. The transcript of BSR0602 was also unregulated in other environmental stresses. These results implied that BSR0602 possibly play important role during infection. To confirm this hypothesis, we isolated total RNA from the spleens of mice infected with *B. melitensis* 16M or 16MΔhfq at different time points post the inoculation. Expression of BSR0602 was determined by qRT-PCR. For 16M, BSR0602 was present at very high levels in infected spleen tissue when compared with *in vitro* condition. The BSR0602 level increase significantly as the infection progresses and peaked at 28 days post the infection, implying that BSR0602 may function at chronic stage of infection (Figure 2C). Thus, BSR0602 was highly activated during host infections and under *in vitro* stress that simulated conditions encountered in hosts’ phagocytes, suggesting its role in the intracellular survival of *B. melitensis*. Compared with that in wild type strain 16M, transcription level of BSR0602 in 16MΔhfq was significantly decreased during *in vivo* infection (Figure 2C), indicating that BSR0602 was also Hfq dependent during infection.

### *gntR* mRNA is a direct target of BSR0602

**Figure 3:**
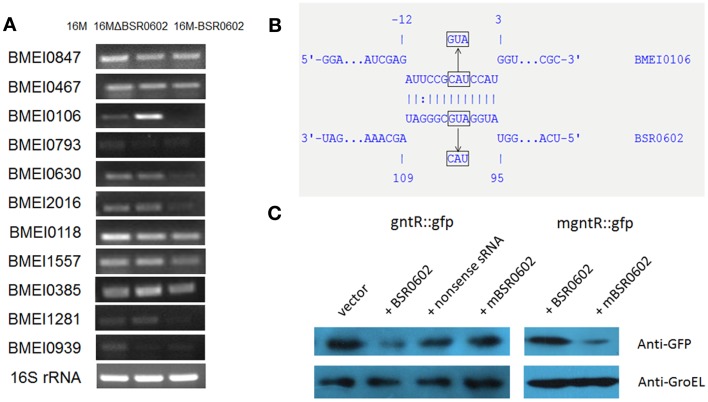
**BSR0602 directly regulates *gntR* mRNA**. **(A)** RT-PCR verification of predicted target mRNA of BSR0602. RNA was isolated from 16M, 16MΔBSR0602, and 16M-BSR0602 and relative transcription of predicted target mRNA genes was quantified and normalized with 16S rRNA. **(B)** Schematic representation of the proposed BSR0602–BMEI0106 interactions and of compensatory base-pair changes. Numbers indicate relative position to the translational start site of BMEI0106 or position downstream of the transcriptional start site of BSR0602. Arrows denote nucleotide substitutions (in box) introduced to BSR0602 and BMEI0106 mRNA. **(C)** Regulation of GntR-GFP reporter fusions by BSR0602. *E. coli* strains carrying only a *gfp* fusion plasmid, or with a combination of both the *gfp* fusion plasmid and sRNA-encoding plasmid, were grown in LB broth, and immunoblot analyses were carried out on total protein lysates to detect levels of GFP or GroEL. Compensatory base pair exchange used for confirming the BSR0602–gntR interaction was also tested by immunoblot analyses. mBSR0602 means mutant BSR0602; mgntR means mutant *gntR*.

Hfq-dependent trans-encoded sRNAs typically act by binding to the 5′-region of target mRNAs, leading to repression of translation initiation and degradation of the mRNA (Nielsen et al., 2011). Thus, target mRNAs identification is a key step to elucidate the role of BSR0602 in the intracellular survival of *Brucella*. Putative mRNA targets of BSR0602 were predicted using TargetRNA, which searches for complementarity between the query sRNA and the 5′ untranslated region (UTR) of mRNAs of annotated ORFs within a given genome (Tjaden, 2008; Bradley et al., 2011). Eleven putative target mRNAs were identified (Table S2 in Supplementary Material). To verify these targets, the expression level of the candidate mRNAs from BSR0602 mutant, BSR0602 overexpression strain and the isogenic wild-type strain 16M were compared using qRT-PCR. Of the 11 putative target mRNAs, expression of BMEI0106 was repressed by BSR0602. Results of qRT-PCR showed that the deletion of BSR0602 increased the level of BMEI0106, while the overexpression of BSR0602 nearly full repressed the expression of BMEI0106 (Figure 3A). Northern blot analysis has the similarly results (Data not shown). These data suggested that BSR0602 negatively regulates BMEI0106, a transcriptional regulator of *gntR* family. Besides BMEI0106, BMEI0630, BMEI2016, BMEI0385, and BMEI1281 seem to be negatively regulated by BSR0602 as the abundance of their mRNA were decreased in the BSR0602 overexpression strains (Figure 3A).

To determine if BSR0602 directly regulates *gntR*, we used a two plasmid system (Corcoran et al., 2012) to investigate the potential interactions between BSR0602 and *gntR* mRNA. First, the RNAhybrid algorithm (Rehmsmeier et al., 2004) was used to predict BSR0602–BMEI0106 interaction (Figure 3B). The predicted targeting region overlaps the RBS of the target mRNA. Thus, the 5′ UTR and the first 15 codes of *gntR* was cloned in-frame with *gfp* into pXG10-SF. Meanwhile, the gene encoding BSR0602 or a nonsense sRNA (BSR0602 sequence in reverse) was cloned into pZE12-luc. *E. coli* Top10 strains were transformed either with the *gfp* fusion construct alone or with a combination of both the *gfp* fusion construct and sRNA-encoding construct. Then, western blot was used to assess the amount of GFP. Prior testing showed that neither BSR0602 nor the nonsense sRNA impact expression of *gfp* in the pXG10-SF (Data not shown), suggesting that the detected expression is specific to the cloned targets. For gntR-gfp fusion, the amount of GFP was strongly reduced in cells co-expressing BSR0602, while the amounts of GFP had no change between the strain carrying the *gfp* fusion and the nonsense sRNA and the “fusion-only” strain (Figure 3C, lanes 2 and 3). The results indicated that BSR0602 directly inhibits *gntR* expression.

To further verify the interactions between BSR0602 and *gntR*, point mutations were introduced to BSR0602 (A_100_U_101_G_102_ > U_100_A_101_C_102_) and *gntR* (C_−5_A_−4_U_−3_ > G_−5_U_−4_A_−3_) to generate mutant m-BSR0602 and m-gntR (Figure 3B). RT-PCR analysis showed that the point mutations do not affect the expression of BSR0602 and *gntR*. Singly, the point mutation of BSR0602 or *gntR* abrogated the repression of *gntR* by BSR0602 (Figure 3C, lanes 4 and 5). However, when the m-BSR0602 and m-gntR were both present, BSR0602-mediated *gntR* repression was restored (Figure 3C, lane 6). These results supported the conclusion that BSR0602 directly regulates *gntR* by forming a short duplex with its mRNA, lending support to *gntR* being a direct target of BSR0602.

### BSR0602 modulate *B. melitensis* intracellular survival through repressing *gntR*

**Figure 4:**
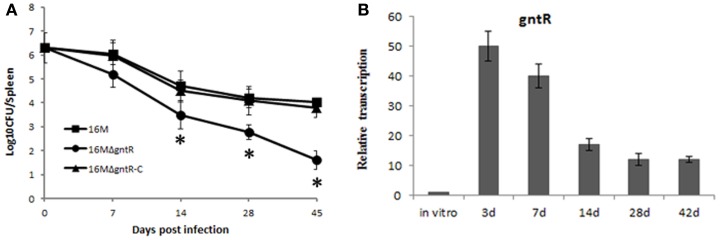
**BSR0602 regulation *Brucella* virulence by targeting *gntR***. **(A)** Contribution of GntR (BMEI0106) to the virulence of *B. melitensis*. Groups of five BALB/c mice were infected intraperitoneally with 2 × 10^6^ CFU of 16M, 16MΔgntR, or 16MΔgntR-C. At 7, 14, 28, and 45 days post inoculation, the spleens were aseptically removed and the CFU were counted by plating serial dilutions on TSA plates. The data were expressed as the mean log_10_ CFU ± SD (*n* = 5). Significant differences between the mutant and parent strain were indicated as follows: *P < 0.001. **(B)** Expression of *gntR* during mouse infection. BALB/c mice (five per time point) were intraperitoneally infected with *B. melitensis* 16M, and total RNA was isolated and subjected to assays of qRT-PCR as described above. The values represent the relative level of *gntR* in 16M recovered from the spleen as compared to the level of *gntR* in *B. melitensis* grown to exponential phase *in vitro*. The SD is indicated by the error bars.

It has been shown that many members of *gntR* family in *Brucella* contribute to its pathogenesis. To test whether *gntR* (BMEI0106) has any influence on *B. melitensis* virulence, a *gntR* (BMEI0106) mutant was constructed and its virulence was evaluated. BALB/c mice were inoculated intraperitoneally with *B. melitensis* and spleen colonization was assessed at different time point postinfection. Compared to the wild-type strain 16M and the complementary strain 16MΔgntR-C, splenic CFU in 16MΔgntR infected mice were significantly reduced (Figure 4A). The results demonstrated that *gntR* was important for the intracellular survival of *B. melitensis*. Additionally, RT-PCR analysis showed *gntR* was highly activated during mouse infection (Figure 4B), again confirming its role in the intracellular survival of *B. melitensis*. Moreover, the expression profile of *gntR in vivo* was contrast to BSR0602 (Figures 2C and 4B), confirming BSR0602 negatively regulates BMEI0106. Hence, BSR0602 may modulate *B. melitensis* intracellular survival through repressing *gntR*.

**Figure 5:**
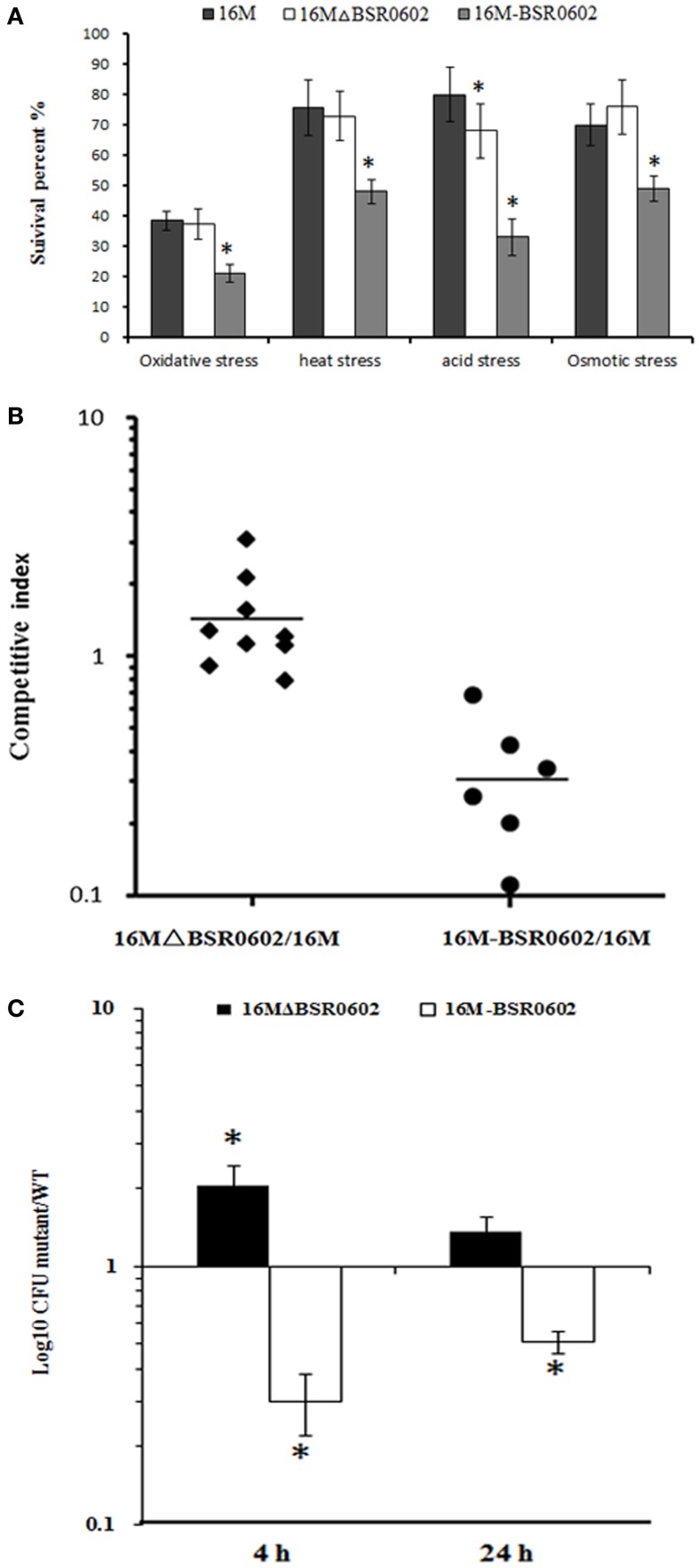
**BSR0602 is involved in *B. melitensis* intracellular survival**. **(A)** Expression of BSR0602 reduces *in vitro* stress resistance of *B. melitensis*. 16M, 16MΔBSR0602, and 16M-BSR0602 were grown in TSB to the early logarithmic phase and then subjected to different stress conditions. After the treatments, the surviving bacteria were enumerated by plating serial dilutions onto TSA plates. Bars represent mean percent survival compared to untreated controls, and error bars represent standard errors of percent survival from three replicates. Asterisks (*) represent significant differences compared with 16M. **(B)** Expression of BSR0602 reduces the number of bacteria in spleens of infected mice. Mice were challenged with BSR0602 mutants and parental 16M (or BSR0602 overexpression strains and parental 16M) in a competitive index model of infection. Twenty-four hours post infection, spleens were removed and the bacterial amount for each strain was determined. Each data point represent a single mouse. **(C)** Expression of BSR0602 reduces intracellular multiplication of *B. melitensis*. Murine macrophage-like cells RAW264.7 were incubated with a 1:1 ratio of 16M to 16MΔBSR0602 or 16M-BSR0602. Data are presented as the log_10_ of the ratios of mutant to wild-type CFU from serial dilutions plated in duplicate and averaged over five wells. Error bars represent SD from the means. The limit of detection was <25 CFU/well.

Our results showed that *gntR* was overproduced in BSR0602 mutant than that in the wild type strain (Figure 3A), so we supposed that BSR0602 mutant perhaps be more virulent than the wild type. To determine whether this is the case, the deletion mutant 16MΔBSR0602 and the overexpression strain 16M-BSR0602 were characterized for changes in virulence related phenotypes. First, 16M, 16MΔBSR0602, and 16M-BSR0602 were tested for their survival capability under various environmental stress conditions that simulate the intracellular environments encountered by *Brucellae* during their dissemination. Compared to the wild strain 16M, the survival of BSR0602 mutant 16MΔBSR0602 under oxidative stress, high-temperature, and osmotic stress did not change significantly (*P* > 0.05) (Figure 5A). The survival percentage of 16MΔBSR0602 only decreased slightly (12%) upon exposure to acidic pH. However, the overexpression strain 16M-BSR0602 was significantly reduced in its ability to survive under all stress conditions (*P* < 0.001). Under acid stress, the survival percentage of 16M-BSR0602 even decreased about 50% compared to the wild-type strain. These data demonstrated that overexpression of BSR0602 could lead to the reduced survival of *B. melitensis in vitro*. Then, competitive assays were performed by intraperitoneally inoculating BALB/c mice with 2 × 10^6^ CFU of an equally mixture of two different strains, (i) wild type (16M) plus ΔBSR0602 mutant (16MΔBSR0602) and (ii) wild type (16M) plus BSR0602 overexpression strain (16M-BSR0602). As shown in Figure 5B, 16M-BSR0602 was out-competed by 16M with the median competitive index (CI; output ratio/input ratio) value being 0.30 at 24 h post infection. The significant diminution of the CI values might not be due to an inability to compete under nutrient-limiting conditions, as the 16M-BSR0602 outcompetes 16M with an *in vitro* CI value of 0.76 in GEM medium (Data not shown). This data demonstrated that the BSR0602 overexpression strain was significantly attenuated when compared to the WT *B. melitensis*. In contrast, the deletion of BSR0602 leads to increased colonization of spleen, suggesting that BSR0602 is a negative regulator of virulence. As the ability to replicate within host phagocytes is essential to the pathogenicity of *Brucella*, we also tested the survival ability of 16MΔBSR0602 and 16M-BSR0602 in macrophages. RAW264.7 macrophages were infected with an equal mixture of (i) 16M plus 16MΔBSR0602 and (ii) 16M plus 16M-BSR0602. The ratio of wild type to BSR0602 overexpression strain reveals a 3.3 and 2.0-log difference in survival at 4 and 8 h of mixed infection, respectively (Figure 5C). The results also indicated that BSR0602 is a negative regulator required for the survival of *Brucella* within host macrophages.

## Discussion

While there has been a rapid increase in identification of bacterial sRNAs over the last few years, the identification of mRNA targets and the study of sRNAs function have proceeded more slowly (Arnvig and Young, 2009). In this study, we identified eight novel sRNAs and systematically analyzed one of them, BSR0602, which modulates the virulence capacities of *B. melitensis*. BSR0602 regulates expression of *gntR*, and that its function is important for *Brucella* intracellular survival and virulence in mice.

By using bioinformatic predictions, a total of 21 putative sRNA candidate genes were predicted in *B. melitensis*. Among them, the transcripts of 15 candidate genes were verified by northern blotting and 8 sRNA genes were transcribed independently of the flanking ORFs. Since no any homologs were found in the Rfam database and small RNA database, all these eight sRNAs were identified as potentially novel sRNA candidates. As our prediction are based on the common features of trans-encoded sRNAs such as the location in IGRs, structure conservation, or sequence similarity between species (Wassarman et al., 2001; Vogel and Wagner, 2007; Waters and Storz, 2009), as well as the existence of promoters and Rho-independent terminators in IGRs, we have identified only a small subset of the total *B. melitensis* sRNAs. While our studies were underway, Dong et al. (2014) reported identification of 129 sRNA candidates using a combination of sRNA prediction programs in *B. abortus* in the 34–434 nt range. Twenty sRNA candidates were chosen to test by RT-PCR and seven could be verified. Although the genomic sequences of *B. melitensis* and *B. abortus* have high similarity, none of our verified sRNAs were predicted by their method. This discrepancy may be due to the different prediction methods and parameters used by the two studies. This also indicated that any bioinformatic prediction methods would suffer false positive and missing of real candidates.

In bacteria, many transacting sRNAs require the RNA chaperone Hfq for their functions (Chao and Vogel, 2010). Although the exact role of Hfq is not fully understood, it seems that the chaperone promotes base-pairing interactions of the sRNA and the mRNA target by increasing the rate of sRNA–mRNA association in addition to protecting the sRNAs from degradation (Mohanty et al., 2004; Brennan and Link, 2007; Koo et al., 2011). In our study, six of eight identified novel sRNA genes showed an Hfq-dependent expression profile, again confirming the important role of Hfq in trans-encoded sRNAs. Northern blot results also evidenced the expression level of sRNA varied between exponential and stationary growth phases. Stationary-phase physiology is of great potential benefit to the *Brucellae* toward their ability to successfully adapt to the harsh environmental conditions encountered in the phagosomal compartment and induce chronic infection (Roop et al., 2003). During residence in host macrophages, the *Brucellae* display stationary-phase physiology. Compared with those actively replicating bacterial, intracellular survival bacteria displaying stationary-phase physiology will be harder to kill by antibiotics. This phenomenon was also observed in the intracellular pathogen *Mycobacterium tuberculosis* (Wayne and Sohaskey, 2001). Three sRNAs were significantly induced in the stationary phase and one of them, BSR0602, was chosen for further analysis. We postulated that the other two sRNA might also play important roles in virulence regulation of *B. melitensis*, which were under investigation in our laboratory.

Many sRNAs have been reported to be associated with bacterial responses to stress (Davis et al., 2005; Abu-Qatouseh et al., 2010; Fantappie et al., 2011). Real-time expression analyses demonstrated that BSR0602 was expressed at a higher level under *in vitro* stresses reminiscent of the environments *B. melitensis* encounters in host macrophages. This suggests that BSR0602 could be associated with *Brucella*’s adaptation to conditions encountered during infection, which was also observed in other pathogens (Arnvig et al., 2011). Furthermore, BSR0602 was transcribed at 16-fold higher level than that *in vitro* at 28 days postinfection, a time point where the infection turn into chronic infection. Thus, BSR0602 was highly expressed *in vitro* under conditions resembling those during infection in host phages, and to even higher levels during infection, indicating a potential contribution to pathogenesis.

A growing number of mRNAs encoding transcription regulators appear to be targets of multiple sRNAs (Battesti et al., 2011; Storz et al., 2011). By combining qRT-PCR with *in silico* target prediction, a global transcription regulator *gntR* (BMEI0106) was identified to be the target for BSR0602. The majority of the regulation by the known trans-encoded sRNAs is negative (Gottesman, 2005; Aiba, 2007). This class of sRNAs can repress mRNA translation by pairing with the ribosome binding site and occluding ribosome access, or can alter mRNA stability by generating duplex molecules, which act as substrates for RNase III or RNase E (Aiba, 2007; Papenfort et al., 2010; Shao and Bassler, 2012). In this study, *gntR* was also negatively regulated by BSR0602, since overexpression of BSR0602 results in nearly full repression of BMEI0106. Results from GFP reporter system demonstrated that BSR0602 directly inhibit the expression of *gntR* mRNA and this direct sRNA–mRNA interactions were further confirmed by compensatory mutant experiments. The location of the hypothesized interaction between BSR0602 and *gntR* indicated that BSR0602 may assert its negative regulatory effect on *gntR* expression by hindering ribosome binding. Many bacterial trans-encoded sRNAs required the RNA chaperone Hfq to stabilize the sRNA–mRNA interaction. BSR0602 was determined to be an Hfq-dependent sRNA both *in vitro* and *in vivo*. Examine the role of Hfq in the BSR0602–gntR interaction will help uncover its full role in post-transcriptional regulation.

GntR family of regulators has been shown to be involved in the regulation of many different biological processes (An et al., 2011; Shafeeq et al., 2013). Many members of *gntR* family in *Brucella* have proved to be associated with the virulence of the pathogen (Haine et al., 2005). Our data confirmed that *gntR* contribute to intracellular survival in spleens of infected mice as 16MΔgntR mutant was rapidly cleared from the spleen in BALB/c mice, which was inconsistent with the results of previously reported (Haine et al., 2005). We propose that this discrepancy may be due to the different methods used to construct the *gntR* mutant. Haine et al. (2005) used the plasmid-tagged mutagenesis (PTM) method, which could test several mutants simultaneously in one animal. However, PTM method was only successful on 80% of the integrative mutants (Haine et al., 2005). *In vivo* transcriptional analysis indicated that *gntR* accumulated to high levels during infection, again confirming its role in pathogenesis. So, BSR0602 may modulate *B. melitensis* intracellular survival through repressing *gntR*. Considering GntR function as a transcriptional regulatory protein in *B. melitensis*, regulation by BSR0602 may be a two-step process in which BSR0602 regulates GntR and then affects transcription of downstream genes.

BSR0602 overexpression strains showed significant decreased survival under all stress conditions when compared with the wild type strain 16M and the BSR0602 deletion mutants. Moreover, high level of BSR0602 impairs bacterial survival both *in vitro* and *in vivo*. Data presented here would be consistent with the suggestion that decreased intracellular survival was due to the significant declined expression levels of *gntR* in BSR0602 overexpression strains. Although many sRNAs result in impaired virulence upon deletion, sRNAs could be negative regulators for bacterial virulence. For example, the deletion of *Listeria monocytogenes* RliB (Toledo-Arana et al., 2009) and *Vibrio cholerae* VrrA (Song et al., 2008) could lead to increased colonization of spleen and intestines, respectively. Moreover, previous reports have shown that overexpression of MTS2822 in *M. tuberculosis* could be lethal (Arnvig and Young, 2012).

The widespread utilization of RNA-based regulation of diverse processes has a number of potential advantages for bacterial (Beisel and Storz, 2010; Mann et al., 2012). Bacterial sRNAs do not translated into proteins or peptides and only occupy a very limited amount of the genome. Thus, they require less energy and reduce metabolic cost. Additionally, regulation conferred by sRNAs often occurs at the post-transcriptional level, which ensures a faster regulation. Unique kinetic regulatory properties and additional levels of regulation displayed by sRNA-mediated regulation are also advantageous compared to protein-based regulation (Levine et al., 2007; Levine and Hwa, 2008; Beisel and Storz, 2011). After identify sRNAs in pathogenesis, the challenging task is to clarify the predominant targets for sRNA regulation and make clear the position of sRNAs in regulatory circuits. Regulation of multiple targets by a single sRNA is common (Papenfort and Vogel, 2010). Thus, BSR0602 may have other targets besides *gntR*. Eleven putative target mRNAs were identified using TargetRNA; however, these predictions need to be further validated. In summary, we have identified a novel sRNA BSR0602 modulating *B. melitensis* intracellular survival and this provide significant insights for unraveling the sRNA-mediated regulatory networks in *B. melitensis*.

## Conflict of Interest Statement

The authors declare that the research was conducted in the absence of any commercial or financial relationships that could be construed as a potential conflict of interest.

## Supplementary Material

The Supplementary Material for this article can be found online at http://www.frontiersin.org/Journal/10.3389/fmicb.2015.00164/abstract

## Footnotes

^1^http://www.isrec.isb-sib.ch/ftp-server/pftools/

^2^http://rfam.sanger.ac.uk

^3^http://biobases.ibch.poznan.pl/ncRNA/
