# Supplementary material for: Identification of a Novel Small Non-Coding RNA Modulating the Intracellular Survival of Brucella melitensis
Source: Front Microbiol. 2015 Mar 19;6:164. doi: 10.3389/fmicb.2015.00164 (PMC4365724; doi:10.3389/fmicb.2015.00164)
Supplement: Supplementary file 1 [file Table_1.DOCX]

Table S1 Primers used in this study

| Primer | Sequence (5’-3’) |
| --- | --- |
| BSR1944-F | AGTGTTCCAGAATCGTTGATG |
| BSR1944-R | CAATTGCGGCCTTGCAG |
| BSR0742-F | GCAACAGAATCGCCTACAATC |
| BSR0742-R | CGGCTTTTCGCTGTCATTC |
| BSR0709-F | CGGGCATAACGCATTTCG |
| BSR0709-R | TTAATCAATCGCACCAACCAC |
| BSR0653-F | AACGGTCTCCACTGTGAAG |
| BSR0653-R | TTGCTGCTCGGCTGTTAG |
| BSR1350-F | GTCCTTGACTGCGTTACG |
| BSR1350-R | GGCTATCCTTACCATGTCAG |
| BSR1007-F | TCGCCTGTAACGCACTTTATC |
| BSR1007-R | GCCGCTGTAATCCTCATGTC |
| BSR0743-F | TGTAGCTTGACTATGGATGCC |
| BSR0743-R | ACCGCCGCTCCTTCAC |
| BSR0739-F | GCAAATACAGGCTTAGTTGAG |
| BSR0739-R | TGGTGCGAGCAGGAAC |
| BSR0617-F | TTAGTGTCCGCAAGCATGAAC |
| BSR0617-R | CCGAGGGAGCCTGTCTTTC |
| BSR1073-F | TGAATGTTGGGCTGTGGAG |
| BSR1073-R | GCCTCAATGGTGGAACAAC |
| BSR0322-F | AAACCGTTTCACACTTTTCG |
| BSR0322-R | AAAGCCCCGCTCATCTG |
| BSR0201-F | TTTCCGCCGCTTCAAACC |
| BSR0201-R | AACAATAGGCGCGGATCAC |
| BSR1915-F | CGGCTTGATAACAAAATATGG |
| BSR1915-R | AAAAGCCCGCCATTCG |
| BSR0742-F | TCCTGCTACCTGCGATTG |
| BSR0742-R | TCGGTGAGGGCTGGAG |
| BSR0626-F | AAATCGAAAACGGTAGCATTC |
| BSR0626-R | GAAGGCACGCGGGTTG |
| BSR0602-F | CCAATCCCAACTTTTGTCCAG |
| BSR0602-R | GGCGGCGTTTTCGTTTC |
| BSR1141-F | TCACCATAAGAGCAACAACGG |
| BSR1141-R | TCGCCCTGCCAATTGAATAC |
| BSR0437-F | GAATCGGACCAGGGAAAGTG |
| BSR0437-R | CGCTAGGACGGGATAAAGAC |
| BSR1133-F | TCATCGGACGCAGATTGTTTC |
| BSR1133-R | TGTCGGTCGGTATGGTAAGTG |
| BSR0377-F | TTAGAGTTGCCAAGATGCTAG |
| BSR0377-R | AATAGTCGGGCTTTATTCCAG |
| BSR0992-F | ATCACGCAACCCGCAAAAG |
| BSR0992-R | ATCACGCAACCCGCAAAAG |
| BSR0602-N-F | ACGTGGTACCGGTACCACGATATAGGGATTGGAGAAG |
| BSR0602-N-R | ACGTCTCGAGACTCACAATATTTCAATAATTG |
| BSR0602-C-F | ACGTGTCGACTGATCTTGATTCACGATGTTG |
| BSR0602-C-R | ACGTAAGCTTCTGCAGATTACGAAACCATCTGCTCCG |
| pUC19K-F | ATCAGGACATAGCGTTGGC |
| BSR0602-I-R | GTCACCAACTGCCTCTTTGG |
| BMEI0106-N-F | ATCGCTGCTGGATGGTGA |
| BMEI0106-N-R | GACATTCATCCCAGGTGGCGCAGATAATCGGCGACATA |
| BMEI0106-C-F | TCTGGGGTTCGAAATGACCGTGCGTTGTAAATTGTCGT |
| BMEI0106-C-R | CACGGCTTCATTCTGGA |
| BMEI0106-I-R | CGAAGCTCGAAGCCTATGAT |
| BMEI0847-F | GCTTATCCAGCGTTCAGA |
| BMEI0847-R | GCAGCGATGTGGCAAAGA |
| BMEI0467-F | CCTGGCAGAAGGATGACG |
| BMEI0467-F | ATGGGCGATGAGCGAAAT |
| BMEI0106-F | CGAAACGGATTTGGTGGT |
| BMEI0106-R | AACGAAAGGCTCGGCATA |
| BMEI0793-F | CATCAGACCTATACCGAG |
| BMEI0793-R | CACGACAGCGATGACCTT |
| BMEI0630-F | TTTCGGAAACGGTGTTCA |
| BMEI0630-R | GCTGTTCTCGCCCAGGAT |
| BMEI2016-F | GCGGAGGCATTTGCTGAA |
| BMEI2016-R | GGTCGCCAGTCATTTCGT |
| BMEI0118-F | GCCAAATATGTCATCAAG |
| BMEI0118-R | TGCGTCGTCGTAAAGGTC |
| BMEI1557-F | CTGGCTGGAAGATCACGG |
| BMEI1557-R | AGGCAGTTTGCGGAATGT |
| BMEI0385-F | AAACCTGTGCTGGAAATG |
| BMEI0385-R | AGGATCGTCACCACCTTG |
| BMEI1281-F | GTAAAGGCTCGCAAGTCC |
| BMEI1281-R | GAACCTGGCTATCCACAA |
| BMEI0939 | AAAGCGGCATATCCGACA |
| BMEI0939 | ATCGGGCAAGGACATCGA |
| BMEI0106-gfp-F | GCATGCATCATTCCGGTCGTCGTAGC |
| BMEI0106-gfp-R | GCGCTAGCGAGGGGCTTTTCCAGACG |
| BSR0602-ex-F | 5’P-ACTTTTGTCCAGTTGGGC |
| BSR0602-ex-R | GCTCTAGAAAAGGATGCGACCAGACC |
