# Supplementary material for: Identification of a Novel Small Non-Coding RNA Modulating the Intracellular Survival of Brucella melitensis
Source: Front Microbiol. 2015 Mar 19;6:164. doi: 10.3389/fmicb.2015.00164 (PMC4365724; doi:10.3389/fmicb.2015.00164)
Supplement: Supplementary file 2 [file Table_2.DOCX]

Table S2

Putative mRNA targets of BSR0602 identified by TargetRNA

| Locus | NCBI GI | Protein Description | Gene | COG^a^ | Location^b^ | Energy | Pvalue |
| --- | --- | --- | --- | --- | --- | --- | --- |
| BMEI0847 | 17987130 | preprotein translocase subunit SecG | secG | U | C | -14.68 | 0.001 |
| BMEI0467 | 17986750 | coproporphyrinogen III oxidase | - | - | U | -13.44 | 0.003 |
| BMEI0106 | 17986390 | GntR family transcriptional regulator | gntR | K | C | -12.74 | 0.005 |
| BMEI0793 | 17987076 | protease I | - | R | U | -12.45 | 0.006 |
| BMEI0630 | 17986913 | phenazine biosynthesis protein PhzF | phzF | R | C | -11.36 | 0.011 |
| BMEI2016 | 17988299 | hypothetical protein | - | S | C | -10.67 | 0.017 |
| BMEI0118 | 17986402 | integral membrane protein | - | R | CM | -10.02 | 0.023 |
| BMEI1557 | 17987840 | arsenate reductase | arsC | P | C | -9.75 | 0.026 |
| BMEI0385 | 17986668 | membrane-associated alkaline phosphatase | - | S | CM | -9.33 | 0.032 |
| BMEI1281 | 17987564 | dihydroorotase | - | F | C | -9.13 | 0.035 |
| BMEI0939 | 17987222 | hypothetical protein | - | - | U | -8.64 | 0.043 |

a: Abbreviation of cellular role categories of theoretical (http://www.ncbi.nlm.gov/COG/).

b: Abbreviation of cellular location. Protein cellular location was annotated by PSORTb V. 2.0 (http://www.psort.org/).
